# High-frequency laser therapy: a new alternative to physiotherapy in the treatment of cervical disk hernia

**DOI:** 10.3389/fmed.2024.1429660

**Published:** 2024-12-19

**Authors:** Ozlem Kuculmez, Emine Dündar Ahi, Sacide Nur Cosar, Sukran Guzel

**Affiliations:** ^1^Department of Physical Medicine and Rehabilitation, Baskent University Alanya Hospital, Antalya, Türkiye; ^2^Department of Physical Medicine and Rehabilitation, Kocaeli Health and Technology University, Kocaeli, Türkiye; ^3^Department of Physical Medicine and Rehabilitation, Abdurahman Yurtaslan Ankara Oncology Education and Research Hospital, Ankara, Türkiye; ^4^Department of Physical Medicine and Rehabilitation, Ankara Etlik City Hospital, Ankara, Türkiye

**Keywords:** exercise therapy, high-frequency laser therapy, laser, physiotherapy techniques, transcutaneous electrical nerve stimulation

## Abstract

**Introduction:**

High-frequency laser therapy has been increasingly used in several musculoskeletal disorders, but there is still a lack of evidence for the usage of the device in neck pain. This study aimed to compare the effectiveness of physiotherapy, high-frequency laser, and exercise therapy methods in the treatment of pain in cervical disk herniation.

**Methods:**

It was a multicenter, randomized, controlled clinical trial. Patients aged between 18 and 65 with neck pain and a diagnosis of cervical disk hernia were included in the study. Patients with a history of cervical surgery, rheumatism, cancer, or pacemaker were excluded from the study. The patients were randomized into 15 sessions of physiotherapy, high-frequency laser, or exercise therapy groups and evaluated with a range of motion, visual analog score, Neck Disability Index, and Short Form Health Survey-36 before treatment, after treatment, and in 1st and 3rd months. *p* < 0.05 was considered statistically significant.

**Results:**

In total, 150 patients were analyzed. There was a significant improvement in range of motion, visual analog score, Neck Disability Index, and Short Form Health Survey-36 scores in three groups after 3-month follow-up (*p* < 0.05). The improvement was statistically greater in the physiotherapy and high-frequency laser therapy groups (*p* < 0.05), but there was no significant difference between these two groups (*p* > 0.05).

**Discussion:**

The results in the physiotherapy and high-frequency laser therapy groups were better than the exercise group. They may be alternatives to each other in cervical disk hernia treatment.

## Introduction

Neck pain is a frequent musculoskeletal complaint; approximately 70% of people experience neck pain at least once in their lives. The prevalence of neck pain is reported to be 15–50% worldwide, and it has been reported that the frequency has not decreased in the last 10 years (1, 2). Stress, anxiety, cognitive variables, social support, sleep problems, personality, and behavior are risk factors in addition to musculoskeletal disorders (2). The most common reasons for neck pain are myofascial syndrome, cervical spondylosis, and discogenic pain. Cervical disk herniation (CDH) may be detected in both men and women, with an increasing prevalence in the third and fifth decades of their lives. The diagnosis is more common in women, with a rate of 60% (3). The most common symptoms and signs vary depending on the level affected, diameter, and location of the hernia (lateral, central, or foraminal). Neck pain may radiate to the shoulder and arm. Although the most common symptom is neck pain, paresthesia, radicular pain, and loss of strength may also be detected (1). Magnetic resonance imaging (MRI) is the most sensitive method for demonstrating hernias, surrounding soft tissues, and possible root-nerve compression in the diagnosis of CDH (3).

In its treatment, a wide range of modalities including pharmacological treatment and conventional methods such as neck collar, hotpack, ultrasound, transcutaneous electrical nerve stimulation (TENS), traction, vacuum interference, low-level laser therapy, exercise, manual therapy, yoga, interventional injection techniques, and ozone therapy are applied (3). Surgical treatment may be required in patients with surgical indications (4).

Laser therapy is a physical therapy method that uses rays to increase physiological healing processes in the body. The new laser technology, including pulsed pulse technology, was determined to be high-frequency laser therapy, which provides painless therapy and prevents complications such as burning with an advantage of 5–7 cm deep penetration to the tissues. High-frequency laser therapy devices were approved by the FDA in 2005. It has been thought that laser therapy provides improvement with its photochemical [enzymatic activation, increase in adenosine triphosphate (ATP) synthesis, increase in cellular metabolism, increase in pain threshold], photothermal (increase in oxygenation and circulation), and photomechanical (increase in extracellular matrix synthesis, regeneration, lymphatic circulation, and microcirculation and decrease in edema) effects (5, 6). As it has been known that it is more effective than low-intensity laser therapy with its more intense and deeper effect, high-frequency laser therapy has been increasingly used in several musculoskeletal disorders, but there is still a lack of evidence for the usage of the device in neck pain (7, 8).

The aim of this study was to determine the effectiveness of HILT in neck pain originating from cervical disk herniation and compare the effectiveness of exercise, HILT, and conventional physical therapy methods (hotpack, ultrasound, and TENS).

## Materials and methods

A multicenter, randomized, controlled trial was performed on patients suffering from neck pain originating from cervical disk herniation. The study was approved by an ethical committee (03.02.2022, E-94603339-604.01.02-100343) and performed according to the Declaration of Helsinki. A consent form was obtained from all patients. In addition, a clinical trial number (NCT05474625) and approval from the Medical Device Agency (07.06.2021, E-68869993-511.06-452352) were also obtained.

### Patient selection, sampling, and randomization

**Figure 1:**
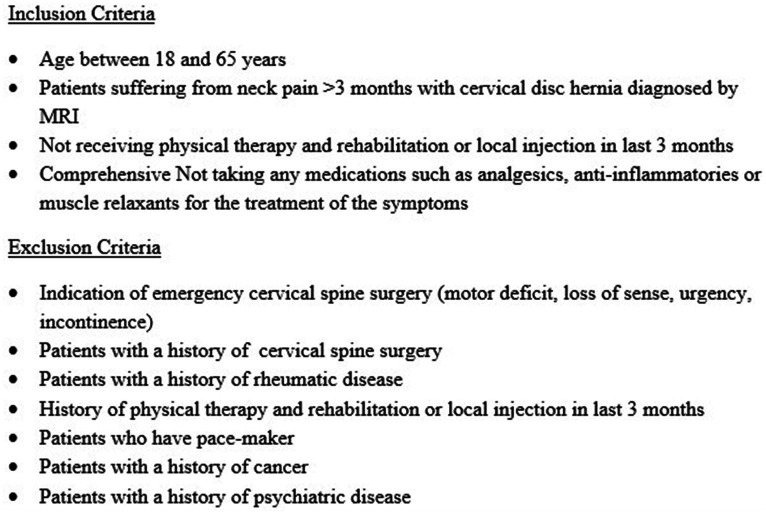
Inclusion and exclusion criteria for the study.

The sample size was calculated using G*Power 3.0.10. (Franz Faul, Universität Kiel, Kiel, Germany) program. Considering an effect size of 0.138 according to the analysis of variance in repeated measurements, it was envisaged to include at least 120 cases (40 for each group) to test the statistical significance of the differences between the groups at a power of 85% and false-positive rate of 5. Power analysis was based on the Neck Disability Index, which was accepted as the primary outcome. Considering that there may be a 20% data loss, 50 patients were included in each group. Non-probability and consecutive sampling methods were used, and randomization was planned according to the stratified randomization method. The patients who accepted to sign consent forms were evaluated regarding inclusion and exclusion criteria, as shown in Figure 1, and patients who met these conditions were randomly separated into three groups (exercise, HILT, and conventional physiotherapy).

### Treatment protocols

#### Exercise

Patients in the exercise group performed 15 sessions of exercise therapy (for 3 weeks on weekdays) including active range of motion exercises, isometric strengthening exercises, and cervical region stretching exercises for 15 min under the supervision of a physiotherapist.

#### High-frequency laser therapy

**Figure 2:**
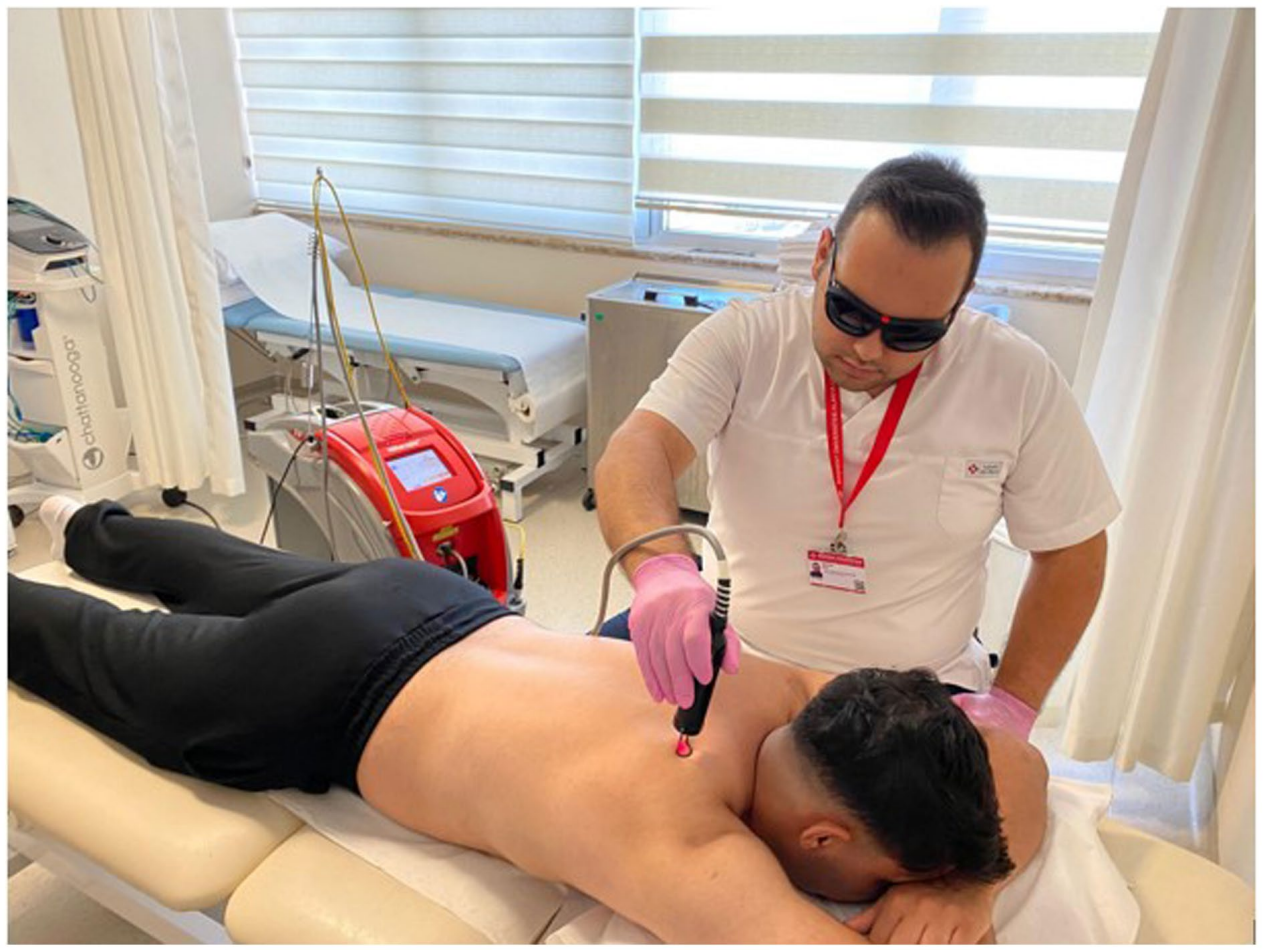
FDA-approved and CE-certified HIRO®3 device (ASA, Arcugnano, Vicenza, Italy) was used for the therapy.

The high-frequency laser therapy group had 15 sessions (for 3 weeks on weekdays) of high-frequency laser and exercise therapy together. For the therapy, an FDA-approved and CE-certified HIRO^®^3 device (ASA, Arcugnano, Vicenza, Italy) was used, as shown in Figure 2. The device has intermittent pulse technology (Nd:YAG laser), and the setting options are pulsed emission (1,064 nm), very high power peaks (3 kW) with short pulse duration (120–150 μs), low frequency (10–30 Hz), and high levels of fluency (360–1780 mJ/cm energy density). Standard 5-mm bright-spot diameter probes and protective glasses were used during the process. The treatment protocol consisted of three stages: the scanning phase (fast scanning of the posterior neck and paravertebral muscles, trapezius, sternocleidomastoid, and intrascapular muscles in transverse and longitudinal directions), the initial phase (scanning of trigger points), and the final phase (slow scanning of the same muscles in the first phase). In total, 2,500 J/cm^2^ (in the scanning phase 1,000 J/cm^2^, the initial phase 500 J/cm^2^, and the final phase 1,000 J/cm^2^) was applied, and the process took 15 min.

#### Conventional physiotherapy

Patients in the conventional physiotherapy group had 15 sessions (for 3 weeks on weekdays) of conventional physiotherapy (TENS, hotpack, and ultrasound) and exercise together. A hotpack (Chattanooga, 15*50 cm) was applied to neck muscles for 15 min. Ultrasound (Cosmogamma mixing 2-combined therapy) was applied with 1 MHz and 1.5 W/cm^2^ doses with ultrasound gel to the right and left cervical paravertebral regions for 4 min, totaling 8 min. TENS (BTL-Italy) was performed in conventional mode with 4 pieces of 5 × 5-cm-diameter adhesive electrode placed on the cervical paravertebral region, with 80 Hz frequency and 180 ms current for 20 min.

In all centers, a physiotherapist with at least 5 years of experience was assigned for this study, and all applications and exercises were performed by the same physiotherapist.

### Evaluation of patients

Patients were evaluated before treatment, after treatment, in the one month after the therapy and three months after the therapy controls with a range of motion (ROM), the Visual Analog Scale (VAS), the Neck Disability Index (NDI), and the Short Form Health Survey-36 in all centers by the same physiatrist.

The ROM of the cervical spine was determined using a goniometer as flexion, extension, right rotation, left rotation, right lateral flexion, and left lateral flexion. Patients were controlled for correct posture, and all measurements were repeated twice. Normal ROM values for the cervical spine were revealed as flexion 60, extension 75, right rotation 85, left rotation 85, right lateral flexion 45, and left lateral flexion 45 degrees, as determined in the study by Thoomes-de Graaf et al. (9).

The general pain status of the patients was evaluated using VAS. The scale is 10 cm long; the left extreme point defines no pain, and the right extreme point defines the most severe pain. Patients determined the severity of the pain they felt with a point on this scale. The distance between the point determined by the patient and the left extreme point was measured in cm, and the numerical value found between 0 and 10 was accepted as the patient’s pain intensity (10).

The Neck Disability Index was developed by Dr. Howard Vernon in 1980, and a Turkish adaptation version was published in 2012 (11, 12). In this questionnaire, pain (from 0 score/no pain to 5 worst pain) and daily activities (from 0 score/no limitation to 5 maximal limitations) such as working, driving, lifting, sleeping, concentration, reading, and recreational activities were evaluated. A total score of 0 means no disability, and a score of 50 means maximal disability.

The SF-36 is one of the most frequently used quality-of-life scales in healthcare studies, which has been validated in Turkish (13). It consists of simple questions on nine subscales, such as physical functioning (PF), role limitations due to physical health (PH), role limitations due to emotional problems (EP), fatigue (F), emotional wellbeing (EW), social functioning (SF), pain (P), general health (GH), and health change (HC). High scores on all subscales of the SF-36 reflect better quality of life, and reduced scores indicate a decrease in quality of life.

Primary outcome was accepted as NDI; secondary outcomes were revealed as SF-36, VAS, and ROM.

### Statistical analysis

The Statistical Package for the Social Sciences (SPSS) version 22.0 for Windows was used for statistical analysis (IBM Corp., Armonk, NY, United States). The normality of continuous values was tested using the Shapiro–Wilk test, and it was found that the values did not follow a normal distribution. As the values were not normally distributed, they were expressed as median (minimum–maximum) and categorical variables as frequency and percentage. The Friedman’s test was used for two-way analysis of variance for repeated measures, was used. When the Friedman test showed that the medians were not equal, the Wilcoxon test was used as a *post-hoc* multiple comparison method for pairwise comparisons (*p* < 0.017). One-way analysis of variance in independent groups was performed with the Kruskal–Wallis test. When the Kruskal–Wallis test showed that the medians were not equal, *post-hoc* multiple comparisons were performed using the Mann–Whitney *U*-test and evaluated using Bonferroni correction (*p* < 0.017). An overall 5% type-I error level was used to infer statistical significance.

## Results

**Figure 3:**
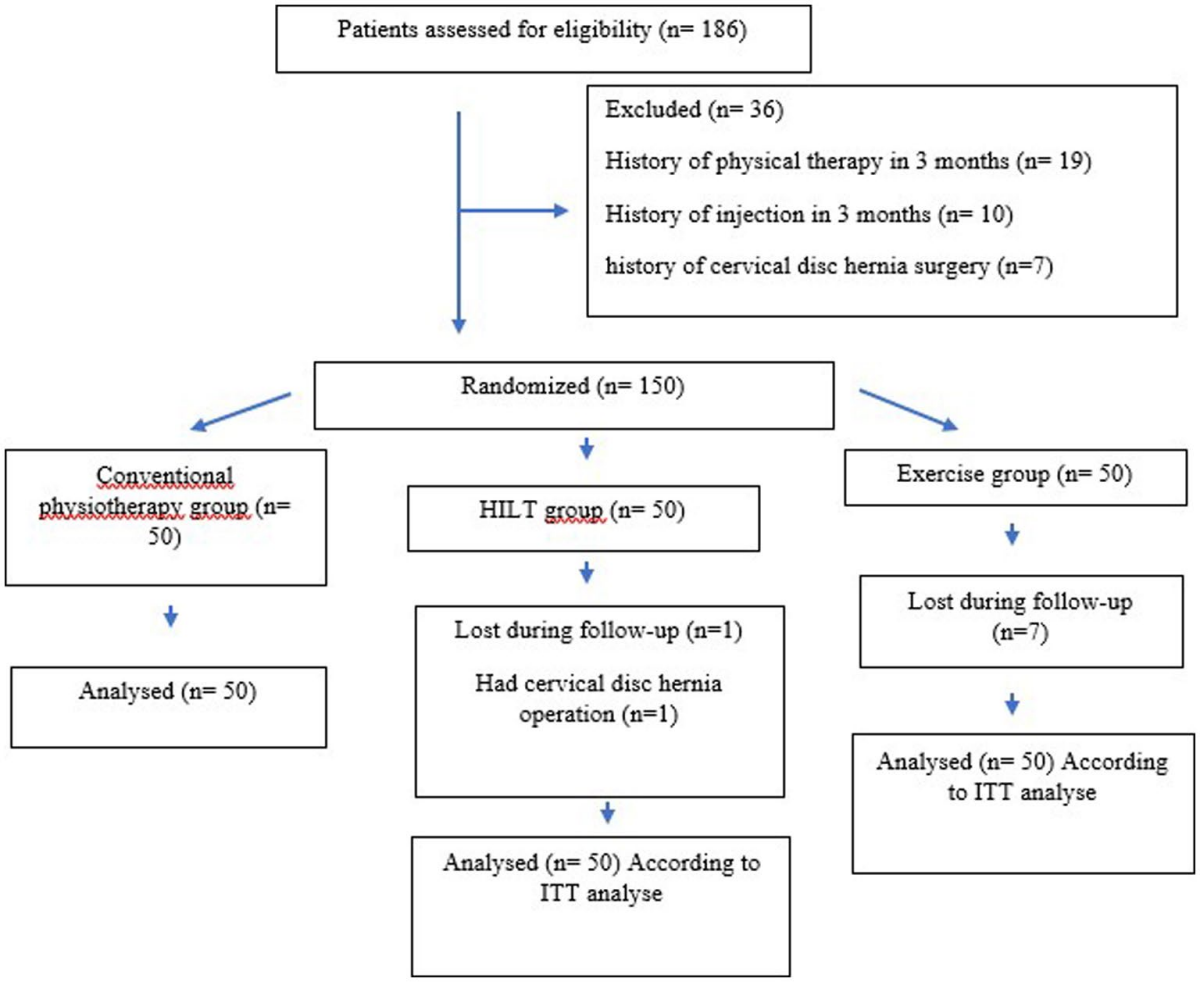
CONSORT (Consolidated Standards of Reporting Trials) flow diagram.

In total, 186 patients were evaluated, and 150 patients who met inclusion criteria were included in this study. Although eight patients (seven patients in the exercise group and one patient in the high-frequency laser therapy group) were lost in the follow-up period and a patient in the high-frequency laser therapy group had operation, a total of 150 patients (50 patients in each group) were analyzed according to intention-to-treat (ITT) analysis. A CONSORT (Consolidated Standards of Reporting Trials) flow diagram is shown in Figure 3. The demographic values of the groups were similar. Overall, 54.7% of the patients were women (*n* = 82) and 45.3% of the patients were men (*n* = 68), with a mean age of 45.37 ± 1.11 years, a body mass index of 25.81 ± 0.33 (kg/m^2^), and a cervical pain duration of 10.10 ± 0.63 months. There was no statistical difference between the groups in terms of age, gender, BMI, and pain duration (*p* > 0.05). The majority of the patients [39.3% (*n* = 59)] had C4–5-level disk hernias, followed by 25.3% (*n* = 38) C3–4 level, 22.7% (*n* = 34) C2–3 level, 10.7% (*n* = 16) C5–6 level, 1.3% (*n* = 2) C1–2 level, and 0.7% (*n* = 1) C6–7 level. In addition, 22% (*n* = 33) of patients had bulging, 62% (*n* = 93) had protrusion, and 16% (*n* = 24) had extruded disk herniation.

After treatment, median values of extension (*p* = 0.005), right (*p* = 0.015), and left rotation (*p* = 0.009) were found to be higher in the high-frequency laser therapy group when compared to the exercise group. The median values of extension (*p* = 0.015), right (*p* = 0.008), and left rotation (*p* = 0.001) after treatment were found to be statistically higher in the physiotherapy group when compared to the exercise group. In all groups, a statistically significant difference was found in median cervical ROM values after treatment and in the 1st month control (*p* < 0.017). Only in the physical therapy group, median cervical ROM values in 3rd month control were found to be statistically significantly higher than before the treatment (*p* = <0.017). The median and interquartile range (IQR) of ROM values are shown in Table 1.

**Table 1:** Change in range of motion during 3-month follow-up.

Flexion	Pretreatment median (IQR)	Posttreatment median (IQR)	1st month median (IQR)	3rd month median (IQR)	*p*-value^a^
Physiotherapy	45 (10)	60 (10)	60 (10)	60 (10)	**0.000** ^*^
HILT	50 (15)	60 (10)	60 (6)	55 (10)	**0.000** ^*^
Exercise	50 (10)	55 (10)	60 (10)	55 (10)	**0.000** ^*^
*p*-value^b^	0.245	0.135	0.436	0.587	

Extension	Pretreatment median (IQR)	Posttreatment median (IQR)	1st month median (IQR)	3rd month median (IQR)	*p*-value^a^
Physiotherapy	50 (15)	60 (25)	60 (25)	60 (25)	**0.000** ^*^
HILT	50 (30)	60 (25)	60 (25)	55 (30)	**0.001** ^*^
Exercise	50 (15)	55 (15)	55 (15)	50 (20)	**0.011** ^*^
*p*-value^b^	0.515	**0.010** ^*^	0.03	0.032	

Right bending	Pretreatment median (IQR)	Posttreatment median (IQR)	1st month median (IQR)	3rd month median (IQR)	*p*-value^a^
Physiotherapy	35 (10)	45 (10)	45 (5)	40 (5)	**0.000** ^*^
HILT	35 (15)	45 (6)	45 (6)	40 (10)	**0.000** ^*^
Exercise	35 (10)	40 (10)	45 (10)	40 (10)	**0.000** ^*^
*p*-value^b^	0.3	0.174	0.639	0.624	

Left bending	Pretreatment median (IQR)	Posttreatment median (IQR)	1st month median (IQR)	3rd month median (IQR)	*p*-value^a^
Physiotherapy	35 (10)	45 (10)	45 (10)	45 (5)	**0.000** ^*^
HILT	35 (15)	45 (5)	45 (6)	45 (10)	**0.000** ^*^
Exercise	35 (15)	40 (10)	45 (5)	40 (10)	**0.000** ^*^
*p*-value^b^	0.587	0.257	0.855	0.653	

Right rotation	Pretreatment median (IQR)	Posttreatment median (IQR)	1st month median (IQR)	3rd month median (IQR)	*p*-value^a^
Physiotherapy	45 (40)	75 (20)	75 (20)	75 (23)	**0.000** ^*^
HILT	60 (40)	75 (16)	75 (16)	75 (20)	**0.000** ^*^
Exercise	65 (25)	70 (20)	75 (20)	70 (15)	**0.000** ^*^
*p*-value^b^	0.033	**0.016** ^*^	0.415	0.049	

Left rotation	Pretreatment median (IQR)	Posttreatment median (IQR)	1st month median (IQR)	3rd month median (IQR)	*p*-value^a^
Physiotherapy	45 (40)	75 (33)	75 (30)	75 (25)	**0.000** ^*^
HILT	60 (40)	75 (15)	75 (16)	75 (25)	**0.000** ^*^
Exercise	60 (25)	70 (25)	75 (20)	70 (15)	**0.000** ^*^
*p*-value^b^	0.124	**0.002** ^*^	0.219	**0.016** ^*^	

^*^Statistically significant *p* < 0.05. IQR, interquartile range.

a Freidman test.

b Kruskal–Wallis test.

There was no statistically significant difference between the groups in median VAS and NDI values after treatment in 1st month and 3rd month controls (*p* < 0.05). In comparisons before and after the treatment within the groups, a statistically significant improvement was detected in median VAS and NDI values, and the improvement was sustained for 3 months in all groups (*p* < 0.017). Median and IQR VAS scores and NDI scores are shown in Tables 2, 3.

**Table 2:** Change in visual analog score during 3-month follow-up.

VAS	Pretreatment median (IQR)	Posttreatment median (IQR)	1st month median (IQR)	3rd month median (IQR)	*p*-value^a^
Physiotherapy	8 (4)	3 (3)	2 (3)	2 (3)	**0.000** ^*^
HILT	7 (4)	3 (2)	3 (2)	2 (2)	**0.000** ^*^
Exercise	6 (3)	3 (3)	3 (3)	3 (3)	**0.000** ^*^
*p*-value^b^	0.032	0.749	0.401	0.027	

^*^Statistically significant *p* < 0.05. IQR, interquartile range.

a Freidman test.

b Kruskal–Wallis test.

**Table 3:** Change in Neck Disability Index during 3-month follow-up.

NDI	Pretreatment median (IQR)	Posttreatment median (IQR)	1st month median (IQR)	3rd month median (IQR)	*p*-value^a^
Physiotherapy	42 (30)	22 (16)	18 (17)	26 (25)	**0.000** ^*^
HILT	36 (15)	18 (19)	18 (16)	24 (25)	**0.000** ^*^
Exercise	32 (22)	20 (18)	20 (16)	22 (12)	**0.000** ^*^
*p*-value^b^	**0.012** ^*^	0.22	633	0.937	

^*^Statistically significant *p* < 0.05. IQR, interquartile range.

a Freidman test.

b Kruskal–Wallis test.

After 3-month follow-up, there was significant improvement in all SF-36 subscales (physical functioning, role limitations due to physical health, role limitations due to emotional problems, fatigue, emotional wellbeing, social functioning, pain, general health, and health change) (*p* < 0.05). In *post-hoc* analysis, it was shown that improvement in physical functioning started after treatment and was sustained for 3 months. Emotional wellbeing improved after the 1st month. Statistical significant improvement in other parameters was detected in the 3rd month control. Median and IQR SF-36 scores are shown in Table 4. A statistically significant difference was found between median values of SF-36 scores after treatment in 1st month and 3rd month controls in the physiotherapy group (*p* = 0.012), high-frequency laser therapy group (*p* = 0.007), and exercise group (*p* = 0.002), respectively. In a pairwise comparison between high-frequency laser therapy and physiotherapy groups, no statistically significant difference was found between median values of physical function scores after treatment (*p* = 0.734) and in 1st month (*p* = 0.374) and 3rd month controls (*p* = 0.543). When the physiotherapy and exercise group’s median values of physical function scores were compared, a statistically significant difference was found in the physiotherapy group after the treatment (*p* = 0.015) and in 3rd month control (*p* = 0.007). When high-frequency laser therapy and the exercise group’s median values of physical function scores were compared, a statistically significant difference was found in the high-frequency laser therapy group after treatment (*p* = 0.006) and in 1st month (*p* = 0.002) and 3rd month controls (*p* = 0.001). A statistically significant difference was found between median values of the emotional wellbeing scores of SF-36 in the 1st month control in physiotherapy, high-frequency laser therapy, and exercise groups, respectively (*p* = 0.010). In pairwise comparison between the groups, median emotional wellbeing values were found to be statistically significantly higher only in the high-frequency laser therapy group (*p* = 0.003). A statistically significant difference was found in all of the groups when pre-treatment and post-treatment median values of SF-36 subscores were compared (*p* < 0.017) except for the role limitation due to emotional problems and general health scores (*p* > 0.017).

**Table 4:** Change in Short Form Health Survey-36 score during 3-month follow-up.

Physical functioning	Pretreatment median (IQR)	Posttreatment median (IQR)	1st month median (IQR)	3rd month median (IQR)	*p*-value^a^
Physiotherapy	65 (20)	80 (25)	85 (20)	95 (20)	**0.001** ^*^
HILT	72.50 (15)	85 (15)	95 (20)	95 (20)	**0.001** ^*^
Exercise	70 (35)	75 (30)	80 (30)	80 (30)	**0.001** ^*^
*p*-value^b^	0.066	**0.012** ^*^	**0.007** ^*^	**0.002** ^*^	

PH	Pretreatment median (IQR)	Posttreatment median (IQR)	1st month median (IQR)	3rd month median (IQR)	*p*-value^a^
Physiotherapy	0.00 (25)	75 (100)	100 (100)	100 (100)	**0.001** ^*^
HILT	0.00 (50)	75 (100)	100 (50)	100 (31. 3)	**0.001** ^*^
Exercise	50 (100)	75 (100)	100 (100)	100 (50)	**0.001** ^*^
*p*-value^b^	**0.001** ^*^	0.515	0.729	0.899	

EP	Pretreatment median (IQR)	Posttreatment median (IQR)	1st month median (IQR)	3rd month median (IQR)	*p*-value^a^
Physiotherapy	0.000 (33. 3)	66.70 (100)	100 (100)	100 (100)	**0.001** ^*^
HILT	0.000 (66. 7)	66.70 (100)	100 (33. 3)	100 (33. 3)	**0.001** ^*^
Exercise	66.70 (100)	100 (100)	100 (100)	100 (100)	**0.012** ^*^
*p*-value^b^	**0.001** ^*^	0.461	0.548	0.518	

Energy fatigue	Pretreatment median (IQR)	Posttreatment median (IQR)	1st month median (IQR)	3rd month median (IQR)	*p*-value^a^
Physiotherapy	30 (30)	50 (20)	50 (28)	55 (23)	**0.001** ^*^
HILT	42.5 (30)	50 (25)	55 (33)	57.50 (26)	**0.001** ^*^
Exercise	45 (25)	50 (15)	55 (15)	50 (10)	**0.002** ^*^
*p*-value^b^	0.168	0.927	0.294	0.367	

Emotional wellbeing	Pretreatment median (IQR)	Posttreatment median (IQR)	1st month median (IQR)	3rd month median (IQR)	*p*-value^a^
Physiotherapy	46 (40)	56 (16)	56 (24)	64 (24)	**0.001** ^*^
HILT	48 (36)	56 (25)	68 (24)	68 (29)	**0.001** ^*^
Exercise	52 (24)	56 (28)	52 (32)	52 (32)	**0.005** ^*^
*p*-value^b^	0.346	0.859	**0.010** ^*^	0.074	

Social functioning	Pretreatment median (IQR)	Posttreatment median (IQR)	1st month median (IQR)	3rd month median (IQR)	*p*-value^a^
Physiotherapy	50 (37.5)	68.75 (25)	62.50 (25)	75 (25)	**0.001** ^*^
HILT	50 (37.5)	75 (25)	75 (12.5)	78 (28.13)	**0.001** ^*^
Exercise	50 (37.5)	62.50 (25)	62.50 (37.5)	75 (37.5)	**0.001** ^*^
*p*-value^b^	0.691	0.936	0.299	0.491	

Pain	Pretreatment median (IQR)	Posttreatment median (IQR)	1st month median (IQR)	3rd month median (IQR)	*p*-value^a^
Physiotherapy	22.5 (12.5)	77.50 (28.1)	77.50 (26.3)	67.50 (31.25)	**0.001** ^*^
HILT	22.5 (26.9)	77.5 (35)	73.75 (22.5)	67.50 (22.50)	**0.001** ^*^
Exercise	45 (37.5)	67.50 (32.5)	67.50 (32.5)	55 (22.5)	**0.001** ^*^
*p*-value^b^	**0.004** ^*^	0.246	0.098	0.071	

General health	Pretreatment median (IQR)	Posttreatment median (IQR)	1st month median (IQR)	3rd month Median (IQR)	*p*-value^a^
Physiotherapy	50 (20)	60 (15)	60 (20)	60 (15)	**0.001** ^*^
HILT	52.5 (30)	60 (30)	67.50 (21)	70 (20)	**0.002** ^*^
Exercise	50 (15)	60 (20)	60 (20)	60 (20)	**0.001** ^*^
*p*-value^b^	**0.036** ^*^	0.366	0.417	0.908	

Health change	Pretreatment median (IQR)	Posttreatment median (IQR)	1st month median (IQR)	3rd month median (IQR)	*p*-value^a^
Physiotherapy	25 (0)	50 (50)	50 (50)	50 (50)	**0.001** ^*^
HILT	25 (25)	50 (6)	50 (50)	50 (25)	**0.001** ^*^
Exercise	25 (25)	50 (50)	50 (0)	50 (25)	**0.001** ^*^
*p*-value^b^	**0.007** ^*^	0.966	0.837	0.278	

^*^Statistically significant *p* < 0.05. IQR, interquartile range; PH, role of limitations due to physical health; EP, role of limitations due to emotional problems.

a Freidman test.

b Kruskal–Wallis test.

## Discussion

After 15 sessions of conventional physiotherapy (TENS, hotpack, ultrasound), high-frequency laser therapy, and exercise therapy, we observed statistically significant improvement in pain, functional status, and quality of life in all groups at 3-month follow-up. It was determined that the most significant improvement was in the physiotherapy and high-frequency laser therapy groups, but there was no significant difference between these groups.

TENS, one of the conventional physiotherapy methods, is a non-invasive method that was approved by the FDA in 1972 and has been used safely in physical therapy and rehabilitation. A-alpha and beta fibers carrying the proprioception senses are selectively stimulated by TENS, and this stimulation creates facilitation in substantia gelatinosa at the medulla spinalis level, causing inhibition of the fibers that transmit pain sensation in the presynaptic region. It has been reported to be effective in reducing muscle spasm, inflammation, and pain due to this effect (14). Miao et al. (15) found that TENS was more effective than placebo in 124 patients with cervical spondylosis in their randomized controlled trial (RCT) at 3-month follow-up, but there is no RCT about TENS in patients with cervical disk hernia. In the Cochrane database, very low evidence was detected for TENS to be superior to sham and effective in chronic neck pain (14). In another Cochrane database, it was argued that electrotherapy may be more effective than placebo, but there is not enough evidence that therapy agents are superior to each other (16). Rampazo et al. (17) determined that TENS combined with other interventions seemed to be effective and useful in their meta-analysis. The results of the current study support the meta-analysis as interventions of TENS, hotpack, and ultrasound were combined, and clinical improvement (pain, disability, and quality of life) was detected.

Ultrasound therapy is frequently applied in the treatment of acute or chronic pain and musculoskeletal diseases. Ultrasound increases tissue flexibility and blood flow with its thermal effect, provides pain modulation and a mild anti-inflammatory response, and reduces joint stiffness and muscle spasm. The therapeutic range of ultrasound waves is between 0.75 and 3.3 MHz (5). Although there is insufficient evidence about its efficacy alone in neck pain, it has been reported that it may be preferred together with other agents (18–20). Although we could not find any study that used hotpack alone or in contrast to placebo, the current study determined improvement in pain, disability, and quality of life in the conventional physiotherapy group and supported the idea of using TENS, hotpack, and ultrasound together.

In exercise therapy, strengthening exercises, mobility exercises, posture exercises, stabilization exercises, and proprioceptive exercises may be preferred (21). An ideal exercise program prescribed for cervical region problems should be specific and consist of three phases (light exercises activating deep cervical muscles, strengthening deep postural muscles, and increasing intensity of exercises) (22, 23). Although the ideal exercise program has been tried to be defined, there is not enough evidence for its effectiveness in neck pain treatment alone. Miyamoto et al. (24) found exercise therapy to be cost-effective in low back pain but not in neck pain when compared to other interventions in their meta-analysis. In the current study, the exercise program consisted of ROM, strengthening exercises, and stretching exercises as suggested and was found clinically effective but not as effective as conventional physiotherapy and high-frequency laser therapy methods.

Laser therapy is another conventional method that has been frequently used in patients with neck pain (25). In recent years, pulsed pulse technology has been added to low laser therapy, and it has been aimed at applying safer treatment to deeper tissues with deep impact and higher efficiency. The treatment was named high-frequency laser therapy and was approved by the FDA in 2005. The therapy supplies pain relief, regeneration, and anti-edema effect in tissues. It has three main pathways for these effects such as photochemical, photothermal, and photomechanical. After lazertherapy application enzimatic activation occures and ATP increases in ATP synthesis are accepted as photochemical effects, increase in circulation, oxygenation, and nutrition are photothermal effects, increase in extracellular matrix formation, cell repair, and regeneration are photomechanical effects, it has been known that the therapy may cause secondary biological effects such as analgesia, anti-edema effect, and biostimulation. As it is applied in three phases (fast scan, trigger point application, and slow scan), new devices have navigation technology and faster programs that work spontaneously without the help of the therapist (26, 27).

Haładaj et al. (28) compared the Saunders traction device and high-frequency laser therapy in 174 patients with cervical spondylosis in their RCT. They evaluated patients with VAS and NDI after therapy and after 4 weeks. They found high-frequency laser therapy superior in 4th week control. Alayat et al. (29) compared high-frequency laser therapy + exercise and placebo + exercise in 60 patients with chronic neck pain. They found better results in ROM, VAS, and NDI after 6 weeks in high-frequency laser therapy + exercise groups. Venosa et al. (30) performed an RCT on 84 patients with cervical spondylosis, and they compared 12 sessions of high-frequency laser therapy + exercise and US + TENS + exercise groups. They determined that both groups had improvement in VAS, ROM, and NDI in 4 weeks, but high-frequency laser therapy + exercise groups were more effective than US + TENS + exercise group after 4 weeks. Yilmaz et al. (31) performed an RCT in 40 patients with cervical disk herniation. They compared high-frequency laser therapy + exercise and US + TENS + exercise before and after 20 sessions (4 weeks). Both groups were found to be effective in ROM, VAS, and neck pain and disability scale, and it was thought that they may be used as an alternative to each other. A study by Yilmaz et al. (31) has only a small sample and does not have any control group. Our study has a bigger sample size and three groups (high-frequency laser therapy, physiotherapy, and exercise group as a control group). Because of these issues, although the study found similar results, the findings may be revealed as much more valuable. İnce et al. (32) randomized 90 patients to laser therapy + exercise, placebo + exercise, and exercise groups. They followed the patients for 12 weeks, evaluated the patients using VAS, neuropathic pain assessment, radicular pain assessment, functional activity assessment, health-related quality of life assessment, and ROM, and found much more improvement in the laser therapy + exercise group. After these developments, it has been detected that HILT improves pain, disability, and ROM, and the therapy may be used in neck pain with a moderate level of evidence by Xie et al. (33) and de la Barra Ortiz et al. (34).

In the current study, patients took 15 sessions of US + TENS + hotpack + exercise, high-frequency laser therapy + exercise, and only exercise therapy. Improvement in ROM, VAS, NDI, and SF-36 score in all groups was detected in all groups in 3-month follow-up. US + TENS + hotpack + exercise and high-frequency laser therapy + exercise groups improved more than the exercise group, and there was no statistical difference between these two groups. The results support the findings of the study by Yilmaz et al. (31) which shows these two therapies may be alternatives to each other. Other studies generally were performed in patients with cervical spondylosis and chronic neck pain; they had a small sample size, a short duration for follow-up, and no data about quality of life except İnce et al. (32) study. Performing an RCT in three groups (including only the exercise group) with a larger sample size and a 3-month follow-up duration, including the SF-36 for quality of life, is a strength of the study. The limitations of this study are as follows: the study did not use algometer, neuropathic pain assessment, or radicular pain assessment for evaluating the pain. The main limitation of the study is not including a placebo laser protocol. Furthermore, the number of sessions, dosage, and application duration of high-frequency laser therapy remain unclear in the literature. Therefore, there is still a need for multicenter randomized controlled studies with a larger sample size and a longer duration of follow-up. We need further studies for indication, time, and dosage standardization for high-frequency laser therapy. As new high-frequency laser therapy devices that have navigation systems and supply shorter durations for treatment are produced, we also need comparative studies to determine standard methodology for the treatment. Furthermore, the patients who have been randomly assigned to the exercise group had a significantly higher pretreatment median Short Form Health Survey-36 score than the other two groups. If less severe patients have been randomly included in the group, the baseline may not be able to represent the actual situation, and this issue may be revealed as another limitation.

## Conclusion

After 15 sessions of therapy and 3 months of follow-up period, significant improvement in ROM, VAS, NDI, and SF-36 scores was detected in US + TENS + hotpack + exercise, high-frequency laser therapy + exercise, and exercise therapy groups. There were better results in the physiotherapy and high-frequency laser therapy groups, but there was no statistical difference between these two groups. Combination therapies may be much more effective than exercise therapy alone. Physiotherapy and high-frequency laser therapy may be alternatives to each other in patients suffering from neck pain and diagnosed with a cervical disk hernia. With these methods, pain relief, improvement in functional status, and quality of life may be gained. Further multicenter studies with a larger sample size and comparative studies with new technology devices are needed.

## Data Availability

The raw data supporting the conclusions of this article will be made available by the authors, without undue reservation.
